# Could automated machine-learned MRI grading aid epidemiological studies of lumbar spinal stenosis? Validation within the Wakayama spine study

**DOI:** 10.1186/s12891-020-3164-1

**Published:** 2020-03-12

**Authors:** Yuyu Ishimoto, Amir Jamaludin, Cyrus Cooper, Karen Walker-Bone, Hiroshi Yamada, Hiroshi Hashizume, Hiroyuki Oka, Sakae Tanaka, Noriko Yoshimura, Munehito Yoshida, Jill Urban, Timor Kadir, Jeremy Fairbank

**Affiliations:** 1 MRC Lifecourse Epidemiology Unit, Southampton General Hospital, Southampton, Hampshire UK; 2 Orthopedic surgery, Wakayama Medical University, Wakayama city, Wakayama prefecture Japan; 3 Orthopedic surgery, Kinan Hospital, Tanabe city, Wakayama prefecture Japan; 4 Department of Engineering Science, University of Oxford, Oxford, UK; 5 Arthritis Research UK/MRC Centre for Musculoskeletal Work and Health, Southampton General Hospital, Southampton, Hampshire UK; 6 Department of Preventive Medicine for Locomotive Organ Disorders, 22nd Century Medical & Research Center, Faculty of Medicine, University of Tokyo, Tokyo, Japan; 7 Department of Orthopedic Surgery, Sensory and Motor System Medicine, Graduate School of Medicine, University of Tokyo, Tokyo, Japan; 8 Department of Preventive Medicine for Locomotive Organ Disorders, 22nd Century Medical and Research Center, University of Tokyo, Tokyo, Japan; 9 Department of Physiology, Anatomy and Genetics (DPAG), University of Oxford, Oxford, UK; 10 Nuffield Department of Orthopaedics, Rheumatology and Musculoskeletal Sciences (NDORMS), University of Oxford, Oxford, UK

**Keywords:** Lumbar spinal stenosis, MRI scans, Automated grading, Repeatability, Validation

## Abstract

**Background:**

MRI scanning has revolutionized the clinical diagnosis of lumbar spinal stenosis (LSS). However, there is currently no consensus as to how best to classify MRI findings which has hampered the development of robust longitudinal epidemiological studies of the condition. We developed and tested an automated system for grading lumbar spine MRI scans for central LSS for use in epidemiological research.

**Methods:**

Using MRI scans from the large population-based cohort study (the Wakayama Spine Study), all graded by a spinal surgeon, we trained an automated system to grade central LSS in four gradings of the bone and soft tissue margins: none, mild, moderate, severe. Subsequently, we tested the automated grading against the independent readings of our observer in a test set to investigate reliability and agreement.

**Results:**

Complete axial views were available for 4855 lumbar intervertebral levels from 971 participants. The machine used 4365 axial views to learn (training set) and graded the remaining 490 axial views (testing set). The agreement rate for gradings was 65.7% (322/490) and the reliability (Lin’s correlation coefficient) was 0.73. In 2.2% of scans (11/490) there was a difference in classification of 2 and in only 0.2% (1/490) was there a difference of 3. When classified into 2 groups as ‘severe’ vs ‘no/mild/moderate’. The agreement rate was 94.1% (461/490) with a kappa of 0.75.

**Conclusions:**

This study showed that an automated system can “learn” to grade central LSS with excellent performance against the reference standard. Thus SpineNet offers potential to grade LSS in large-scale epidemiological studies involving a high volume of MRI spine data with a high level of consistency and objectivity.

## Background

Lumbar spinal stenosis (LSS) is defined as a narrowing of the lumbar canal with encroachment of neural structures by surrounding bone and soft tissue [1, 2]. It is thought to be a degenerative condition increasing in prevalence with age and it can cause severe impairment of mobility by intermittent claudication (leg pains that increase in intensity with walking speed and distance travelled). As a consequence, LSS has been the most frequent indication for spinal surgery in patients over 65 years [3, 4].

Magnetic resonance Imaging (MRI) is the imaging technique of choice in the assessment of patients with symptoms suggestive of LSS, given that it allows the detection of minute changes of the intervertebral discs and ligaments [5, 6]. However, there is to date no consensus as to how to define LSS severity on MRI scans [7] and a number of qualitative approaches have been suggested [8, 9]. Moreover, the relationship between findings on MRI and clinical course is the source of some controversy with several studies suggesting a high prevalence of MRI LSS in asymptomatic subjects [10, 11].

Therefore, to move forward our understanding of the risk factors, causes and natural history of LSS, the Wakayama Spine study was created as a longitudinal epidemiological study of a sample of adults in the general population using MRI scans taken in one mobile unit to a standardised protocol [12]. Qualitative grading of radiographic features of MRI of the lumbar spine is time-consuming, requires the skill of an experienced observer and checks of inter- and intra-observer reliability and can be prone to human error. Therefore, there have been attempts to develop automated systems for grading MRI scans which would be particularly useful if they could repeatably grade large quantities of lumbar MRI data for large-scale longitudinal epidemiological studies. SpineNet is one such automated system [13, 14]. Using a machine-learning approach based upon a convolutional neural network, it has been shown that the system can learn to grade degenerative disc disease as accurately as a radiologist [14]. Therefore, using two sets of axial scans taken as part of the baseline of the WSS, we investigated the ability of the SpineNet system to “learn” to grade central LSS in comparison with the qualitative assessment of the trained surgeon.

## Methods

### Participants

The present study, entitled The Wakayama Spine Study, assessed a sub-cohort drawn from the Research on Osteoarthritis/Osteoporosis Against Disability (ROAD) study, a large-scale, prospective study of bone and joint disease using population-based cohorts in Japan. The detailed profile of the ROAD study is described elsewhere [15], Individuals in this study were recruited from resident registries in 3 communities: an urban region in Itabashi, Tokyo; a mountainous region in Hidakagawa, Wakayama; and a coastal region in Taiji, Wakayama. In total, 3040 people (1061 men and 1979 women) consented to take part in a clinical and genetic study approved by the ethics committees of the University of Tokyo and the Tokyo Metropolitan Institute of Gerontology. Participants completed an interviewer-administered questionnaire [15] that included 400 items covering demographics, lifestyle and occupation. Participants underwent anthropometric measurements and assessments of physical performance.

The Wakayama Spine Study involved a subset of ROAD participants from Hidakagawa and Taiji provinces. Participants aged >21 years were recruited, had no contraindications to undergo MRI scanning (e.g. no sensitive implanted devices including pacemakers, and claustrophobia) could walk to the study site and provided written, informed consent. All subjects underwent total spinal MRI using a pre-defined standard protocol in a mobile unit (Excelart 1.5 T; Toshiba; Tokyo, Japan). MRI was not performed: in the presence of a cardiac pacemaker; claustrophobia or if there were other relevant contraindications. The participants were positioned supine, and those with rounded backs were positioned with triangular pillows under their head and knees. The imaging protocol was: sagittal T2-weighted fast spin echo (FSE) (repetition time (TR): 4000 ms/echo, echo time (TE): 120 ms, field of view (FOV):300 × 20 mm), and axial T2-weighted FSE (TR: 4000 ms/echo, TE:120 ms, FOV: 180 × 180 mm). Axial images were taken at each lumbar intervertebral level (L1/2-L5/S1) parallel to the vertebral endplates.

### Assessment of lumbar spinal stenosis

The severity of LSS was assessed for central canal stenosis from the MRI axial sequences by one experienced orthopaedic surgeon (YI). The severity of central canal LSS was qualitatively graded on the axial images as: none; mild - narrowing of the normal area by one third or less; moderate–narrowing of the normal area by between one-third and two-thirds, and; severe as more than two-thirds narrowing [16]. Intra-observer reliability was measured when the observer re-assessed a random sample of 50 of the MRI scans after a period of 1 month, blinded to the original rating obtaining a kappa score of 0.77 (excellent agreement). Moreover, inter-observer variability was compared between the study observer and another experienced orthopaedic surgeon (KN) for a different sample of 50 MRI scans and a kappa of 0.71 was achieved for agreement. None of the MRI scans performed were found to have LSS caused by tumor, inflammatory, or traumatic pathologies.

### Radiological grading by automated readings

The system used was the SpineNet system, which has been described in detail elsewhere [13, 14]. In brief, the system uses T2 MRI input from routine MRI scans acquired from a DICOM file. In the “learning” phase, the SpineNet software is trained to detect radiological features of LSS from the experienced spinal surgeon’s assessments. The software needs to be able to learn without human input and classify multiple radiological features simultaneously. Therefore, the SpineNet system adopts a conventional neural network which can both learn and classify multiple scores at the same time. Using a set of 90% of the available lumbar MRI scans which had been qualitatively assessed as above were used in the training phase. Subsequently, we evaluated the effectiveness of the “trained” system using the 10% remaining MRI scans as an independent sample. Based upon its “learning”, the system graded 5 axial T2 images from L1/2-L5/S1 automatically, grading LSS into 4 grades. In the subsequent “assessment” phase, the grades from the automated test sample were then compared with the pre-defined qualitative assessment made independently by YI.

### Statistical analysis

All statistical analyses were performed using JMP version 10 (SAS Institute Japan, Tokyo, Japan). The variability between YI’s reading and that of the machine was assessed using Lin’s concordance correlation coefficient. Subsequently, the variability was confirmed by a Kappa analysis which dichotomized central LSS comparing grade 3 with grades 0, 1 and 2. We chose to use such comparisons to ensure that the system had as high a rate of specificity as possible rather than risk a high rate of false positives.

## Results

In total, 1011 people in the Wakayama prefecture of Japan were recruited to the Wakayama Spine Study (335 men and 676 women, mean age 66.3 years (range 21–97 years)). After exclusions, complete axial views were available for 4855 lumbar intervertebral levels from 971 participants.

Initially, 90% (*n* = 4365) axial views which had been graded qualitatively by YI were machine learned by the SpineNet system (training set). The remaining 10% (*n* = 490) scans were then graded by the SpineNet system automatically and compared with the qualitative assessment made independently. In total, 76.5% of the total sample were defined with moderate or severe radiographic central stenosis by the spinal surgeon.

**Fig. 1:**
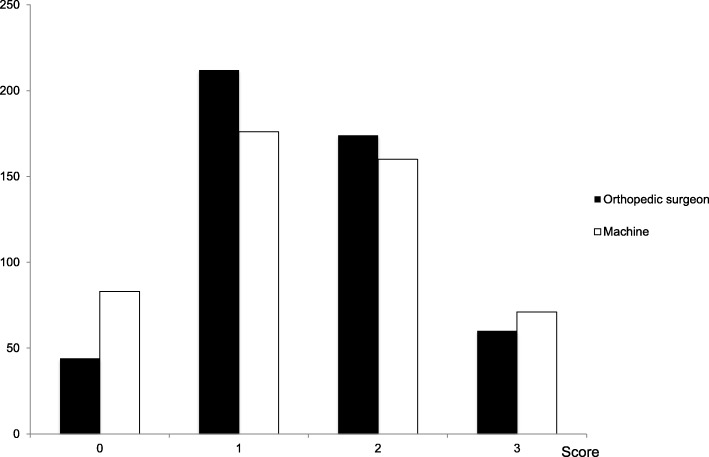
Comparison of the LSS gradings comparing the orthopaedic surgeon with the SpineNet system (*n* = 490 scans)

Figure 1 shows the difference comparing the assessments of YI and the automated readings for each of the 4 grades: none, mild, moderate and severe across the 490 axial views. Overall, the rate of complete agreement in grading (difference of means 0) was 65.7% (322/490) and the reliability calculated with Lin’s correlation coefficient was 0.73. In terms of difference in overall grading, in only 11/490 (2.2%) cases did the assessment of YI and the SpineNet system differ by 2 grades and in only 1/490 scans was there a difference of 3 between the grading assigned by YI and the automated reading.

**Fig. 2:**
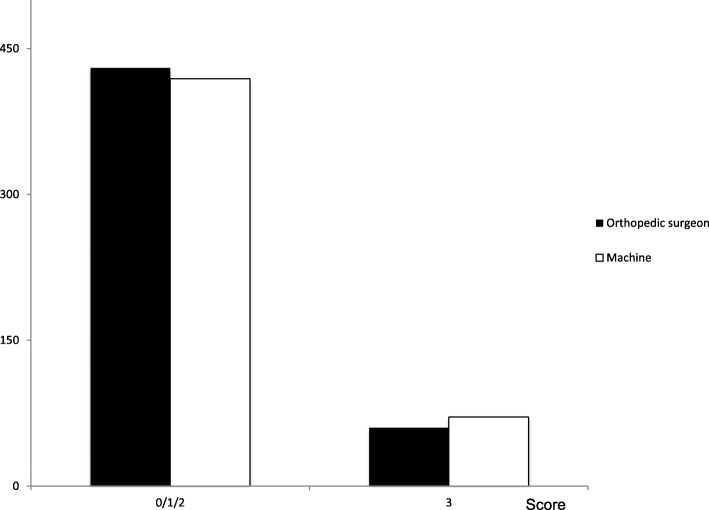
Comparison of the dichotomously classified LSS ratings between the orthopaedic surgeon and SpineNet system

Figure 2 compares the readings of YI with SpineNet when the assessments were compared dichotomously as ‘severe’ vs ‘no/mild/moderate’. Overall, in this analysis, the rate of agreement was 94.1% (461/490) with a kappa of 0.75 for agreement.

## Discussion

We have developed and tested an automated system for classifying MRI features of central LSS, based upon a large number of lumbar MRI scans in a population-based cohort (Wakayama Spine Study). In both analyses (grade 0–4 vs grade 0–4) and grade 4 versus grade 0–3, we found a substantial level of agreement with the automated system (Lin’s concordance correlation coefficient in analysis 1 (good concordance) and kappa in analysis 2 were > 0.7). However, it is noteworthy that there was a high prevalence of moderate /severe LSS in this population sample who were aged on average > 65 years (76.5%). For the system to be useable in large-scale epidemiological studies, we found that where differences in grading were recorded, most only differed by one grade (e.g. mild rated as moderate) (31.8%, 156/490). In only one scan, (0.2%) was there a difference of grading by 3 (i.e. mild rated as severe). This suggests that the software could be used to quickly and reliably assess large amounts of lumbar MRI data without over-classifying cases with LSS, making the technique very suitable for use in epidemiological studies.

These findings need to be considered alongside some limitations. First, the participants in the WSS were a population sample but were not selected at random. To explore their representativeness, we compared the body mass index, smoking status and alcohol intake with general population statistics. We found that the BMI of the WSS participants was almost same as that of general Japanese. However, proportions of current smokers and drinkers in men and that of current drinkers in women were significantly higher in the general Japanese than in the study population, suggesting that they might live healthier lifestyles. This may limit the generalizability of these findings and more validation in a different cohort is recommended.

It is important to note that the gradings generated by the automated system are learned from those presented to it during the training phase so that they depend upon the reference standard. For the purposes of the current study, we chose to use 90% of the available scans for the learning and the remaining 10% for the testing, in line with the protocol used in a similar study grading degenerative disc disease (Jamaludin 14). This does not however allow us to estimate accurately what would be the minimum number of scans needed in order for the automated gradings to attain acceptable levels of accuracy. Moreover, if we used this system, trained on the same dataset and compared them with another assessment of grading scores that was somewhat different from that used here, the grades provided by the automated system would differ accordingly. More research will be required in the future to discover to what extent machine-learned MRI grading can be transferrable on different MRI scanning machines and to what extent the same method could be used across studies. Despite this, the automated system provided gradings which are objective and consistent, making the methodology highly suitable for use in large cohort studies involving spinal MRI.

It is a strength of this study that all MRI scans were performed in the same scanner using a standardised protocol at baseline and were graded by one trained observer who had already been shown to define the grades qualitatively with an excellent level of intra- and inter-observe reliability. SpineNet itself was developed from the Genodisc cohort which used a diversity of scanners [14]. Having shown that the SpineNet system performed very reliably, we will be able to use the system at each follow-up in WSS in order to compare the grades over time in the same individuals and expect a high degree of standardisation.

There is currently controversy about how to optimally classify LSS from spinal MRI. From in vitro experimental studies, Schönström et al. described relative and absolute stenosis as dura cross-sectional area < 100 mm^2^ or < 75 mm^2^ respectively [17]. However, it is technically difficult to apply such methods and calculations in clinical practice, so that these methods have not become widespread. Shizas et al. described a 7-grade qualitative grading based on the morphology of the dural sac measured on axial views and defined by the rootlet/cerebrospinal fluid ratio, however, their average inter-observer agreement was moderate (kappa = 0.44) and the system appeared challenging for a general physician to learn [9]. In practice, clinicians tend to classify the degree of LSS according to a 4 scale qualitative grading as in the current study, but there is no consensus as to the criteria for the 4 gradings, leaving an element of subjectivity. Perhaps because of this, when the variability in assessing LSS by the 4 scale grading comparing 7 observers including 2 orthopedic surgeons, 2 neurosurgeons and 3 radiologists was assessed, the average kappa scores for inter-observer agreement and intra-observer agreement were 0.26 and 0.11 [18], which would be considered ‘fair’ and ‘poor’ agreement respectively according to definitions of Landis and Koch. In particular, the reproducibility was poor, which presents a major problem for interpreting changes in lumbar spine MRI appearances over time in large-scale longitudinal studies. In WSS, an excellent intra- and inter-observer reliability was demonstrated when all readings were undertaken by one clinician (YI). However, to maintain such reliability over follow-up of this number of scans every 3 years, it appears that the automated system offers greater expectations of objective, consistent gradings with lower risk of human error.

## Conclusion

We have shown that MRI grading of central LSS can be predicted with a high degree of reliability and consistency after a period of learning of the reference standard. Such systems are not intended to replace individualized assessment of clinical LSS for making decisions about e.g. surgery [19]. However, these methods have particular promise for use in large-scale longitudinal epidemiological studies involving large quantities of MRI data, studies which are desperately needed if we are to better understand the risk factors, relationship with symptoms and natural history of LSS in the future.

## Data Availability

The datasets used and/or analysed during the current study are available from the corresponding author on reasonable request.

## Abbreviations

BMI: Body mass index
CI: Confidencel interval
LSS: Lumbar spinal stenosis
MRI: Magnetic resonance imaging
OR: Odds ratio
ROAD: Research on Osteoarthritis/Osteoporosis Against Disability
WSS: Wakayama Spine Study
